# The Under-Five Citizen

**Published:** 1919-05-03

**Authors:** 


### THE UNDER-FIVE CITIZEN.

### By A SOCIAL WORKER.

Let us face frankly the official pronouncement on his case as it stands when his home is not one in which nurse-power is available to meet the mother's deficiencies of ability or leisure. The Board of Education's newly-issued regulations simplify the words of the Act which tell us that such schools and classes are to provide for those children of two or five (or a little more) "whose attendance is necessary or desirable for their healthy physical and mental development." Broadly speaking, from the age of two the mental and physical training of all children of the wage-earning classes is taken from that incompetent person their mother, and given into the hands of the trained teacher. On Saturdays and Sundays, and during the night, the mother will be in temporary charge. If she is conscientious, she will no doubt try to make her care of the little child approximate, as far as possible, to the *régime* of the school. It is quite possible that she will come to wish that the child, now shy with her and conscious of unaccustomed surroundings, could be kept at the school during these days also. Is it not even probable that presently the superintendents and teachers will head a movement for the retention of the children of unsatisfactory homes? The drunken mother, alas; it is on Saturday and Sunday that she will be most drunken: the home in which zymotic disease has taken an older child will not be a safe place. Either the nursery-school child must stay there and risk infection, or it will be a danger to others at the school.

Up to the present no provision for such cases has been suggested. Besides these two instances we can foresee a very large number in which the teachers will presently declare that their work is stultified because of the—to borrow a word from the clergy—"Mondayish" condition of the returning children. For five days in the week they have been fed on food "convenient" for them. Their bodily wants have been attended to, not only the school midday dinner is an essential part of the *régime*, with its adjuncts of proper table manners and clean hands and faces, but breakfast and tea are also contemplated for some or all children. Baths—weekly at least—clean clothing, and dry and proper-sized footgear, are also mentioned among probable provisions. Sleep, or at least rest, taken "not sitting but lying down" is also viewed as essential in the school training, and the regularity of physical habit will be steadily preserved during five days of the week. The adequate mother will carry out the intentions of authority on the other two, but the best mother will have difficulties. The child may refuse its dinner, freakishly perhaps, or for better reason. Noise may prevent its sleep, and fretfulness may follow, then she will begin to dread a grave face of embodied authority on Monday morning, despite her best endeavours. As for the inadequate mother, want of practice on five days in the week will not make her more skilful on the two that remain. To a very large class of young girl factory workers, and others whose own childhood was "dragged up," the rapidly succeeding babies come as terrors diminishing by force of habit. The first was a truly dismaying possession, encouragement being chiefly found in the obvious fact that other girls were in like case, and things righted themselves somehow, or one's arms might suddenly be emptied by "churchyard luck." But even to these a sense of responsibility has come with the increase of infant welfare centres and mother-schools. Authority outside the home claims to partial ownership of the child. But if many such girls have found child-care frankly boring, and resorted to the "minder" as soon as possible, so as to get back to the gay insouciance of factory life, the appealing ways of little children have created many an excellent mother, whose sins are only those of ignorance, and whose children on the whole have turned out very well. Avoidable disablements have been lessened in these families by many ways of teaching, such as school hygiene lessons, visits from the "Health Lady." But practical wisdom is gained in all crafts by carrying out work, and the mother who can take her child unwashed and ill-clad to the school on five days a week and receive it back clean, well nourished, and ready for bed, is likely enough to think she is seeking its real good if she too urges that the excellent habits shall not be broken off on two days of every week. "'E's a lot gooder with them nor what 'e is with me," will be a quite truthful and unbiased statement in such a case. But, further, the psychologists and the physiologists are in complete—unusually complete—agreement as to the need for early commencement of right training. At ten minutes after birth, we are taught, the training of nerve-impulses can begin. Why then defer for a couple of years the essential treatment? So we are led on to the climax of Utopian socialism, the immediate removal of the infant to the care of trained experts. "Well," said a working woman to whom this was suggested, "what a home that would be like I can't think. Its the children makes it home."

Perhaps beyond these conditions of to-morrow we can see a further prospect. Will not the nation ultimately see of the travail of its soul and new methods in housing make of the average working-class home a place in which a mother upon whom the promised teaching has had its due effect can fitly rear the working-class child? Experience has convinced us already of the harmfulness of institution life for children, and all our modern policy is in opposition to it, so that boarding out in a real family is held by most educationists to be better than even the Barnardo maximum of sixteen in an artificial home. Yet forty is proposed by the Board's regulations as a satisfactory number, providing always that the ages should be diversified.

Certainly probationers under the age of eighteen are contemplated by the regulations, and since the schools are to be of small size and within walking distance from the home of a child of two, they will necessarily be very numerous, and require, in addition to the superintendent qualified as nurse and teacher, and the teachers who should be interchangeable with other trained women from the upper school, a number of these young learners. It may indeed, before long, become customary, if not compulsory, for elder girls to take a year of this training, especially under the proviso of continuation classes, which is included. When married, will these ex-teachers and probationers whose knowledge of hygiene and domestic science has enabled them to secure proper home premises and organise their life on good lines, be willing to surrender their toddling children to a new generation of young experts and trainers? Is the nursery school, in fact, to be a transient or a permanent feature of our educational system?

